# Effectiveness of a home-based re-injury prevention program on motor control, return to sport and recurrence rates after anterior cruciate ligament reconstruction: study protocol for a multicenter, single-blind, randomized controlled trial (PReP)

**DOI:** 10.1186/s13063-019-3610-2

**Published:** 2019-08-13

**Authors:** Daniel Niederer, Matthias Keller, Andrea Achtnich, Ralph Akoto, Atesch Ateschrang, Winfried Banzer, Alexander Barié, Raymond Best, Andree Ellermann, Andreas Fischer, Daniel Guenther, Mirco Herbort, Jürgen Höher, Maren Janko, Tobias M. Jung, Matthias Krause, Wolf Petersen, Thomas Stoffels, Amelie Stöhr, Frederic Welsch, Thomas Stein

**Affiliations:** 1 Department of Sports Medicine, Goethe University Frankfurt, Ginnheimer Landstraße 39, 40487 Frankfurt am Main, Germany; 2 OSINSTITUT, Munich, Germany; 3 Department for Orthopaedic Sports Medicine, Klinikum rechts der Isar, Munich, Germany; 4 Chirurgisch-Traumatologisches Zentrum, Asklepios Klinik St. Georg, Hamburg, Germany; 5 BG Trauma Center Tübingen, Eberhard Karls University Tübingen, Tübingen, Germany; 6 Department of Preventive and Sports Medicine, Institute for Occupational, Social and Environmental Medicine, Goethe University Frankfurt, Frankfurt, Germany; 7 Clinic for Orthopedics and Trauma Surgery, Center for Orthopedics, Trauma Surgery and Spinal Cord Injury, Heidelberg University Hospital, Heidelberg, Germany; 8 Department of Orthopaedic and Trauma Surgery, Sportklinik Stuttgart, Stuttgart, Germany; 9 Arcus Sportklinik, Pforzheim, Germany; 10 Department of Orthopedic Surgery, Physical Medicine and Rehabilitation, University Hospital, Ludwig-Maximilians-University (LMU), Munich, Germany; 11 Department of Orthopaedic Surgery, Trauma Surgery, and Sports Medicine, Cologne Merheim Medical Center, Witten/Herdecke University, Cologne, Germany; 12 Department of Trauma, Hand and Reconstructive Surgery, University Hospital Muenster, Muenster, Germany; 13 Sports Clinic Cologne at Cologne Merheim Medical Center, Cologne, University of Witten/Herdecke, Cologne, Germany; 14 Department of Trauma, Hand, and Reconstructive Surgery, Goethe-University Frankfurt, Frankfurt am Main, Germany; 15 Center for Musculoskeletal Surgery, Charité-University Medicine Berlin, Berlin, Germany; 16 Department of Trauma, Hand and Reconstructive Surgery, University Medical Center Hamburg-Eppendorf, Hamburg, Germany; 17 Klinik für Orthopädie und Unfallchirurgie, Berlin, Germany; 18 Department of Trauma and Orthopaedic Surgery, Unfallkrankenhaus Marzahn, Berlin, Germany; 19 Orthopedic Surgery Munich, Munich, Germany; 20 Department of Sporttraumatology, Knee, and Shoulder Surgery, Berufsgenossenschaftliche Unfallklinik Frankfurt am Main, Frankfurt am Main, Germany; 21 Department of Orthopaedic Sportsmedicine, University of Tuebingen, Tuebingen, Germany

**Keywords:** Return to sports, Return to play, RTS, Recurrence, Re-injury, ACL, Motor control, Secondary prevention, Therapy, Rehabilitation, Post treatment, Functional outcome

## Abstract

**Background:**

Although anterior cruciate ligament (ACL) tear-prevention programs may be effective in the (secondary) prevention of a subsequent ACL injury, little is known, yet, on their effectiveness and feasibility. This study assesses the effects and implementation capacity of a secondary preventive motor-control training (the Stop-X program) after ACL reconstruction.

**Methods and design:**

A multicenter, single-blind, randomized controlled, prospective, superiority, two-arm design is adopted. Subsequent patients (18–35 years) with primary arthroscopic unilateral ACL reconstruction with autologous hamstring graft are enrolled. Postoperative guideline rehabilitation plus Classic follow-up treatment and guideline rehabilitation plus the Stop-X intervention will be compared. The onset of the Stop-X program as part of the postoperative follow-up treatment is individualized and function based. The participants must be released for the training components. The endpoint is the unrestricted return to sport (RTS) decision. Before (where applicable) reconstruction and after the clearance for the intervention (aimed at 4–8 months post surgery) until the unrestricted RTS decision (but at least until 12 months post surgery), all outcomes will be assessed once a month. Each participant is consequently measured at least five times to a maximum of 12 times. Twelve, 18 and 24 months after the surgery, follow-up-measurements and recurrence monitoring will follow. The primary outcome assessement (normalized knee-separation distance at the Drop Jump Screening Test (DJST)) is followed by the functional secondary outcomes assessements. The latter consist of quality assessments during simple (combined) balance side, balance front and single-leg hops for distance. All hop/jump tests are self-administered and filmed from the frontal view (3-m distance). All videos are transferred using safe big content transfer and subsequently (and blinded) expertly video-rated. Secondary outcomes are questionnaires on patient-reported knee function, kinesiophobia, RTS after ACL injury and training/therapy volume (frequency – intensity – type and time). All questionnaires are completed online using the participants’ pseudonym only.

Group allocation is executed randomly. The training intervention (Stop-X arm) consists of self-administered home-based exercises. The exercises are step-wise graduated and follow wound healing and functional restoration criteria. The training frequency for both arms is scheduled to be three times per week, each time for a 30 min duration. The program follows current (secondary) prevention guidelines.

Repeated measurements gain-score analyses using analyses of (co-)variance are performed for all outcomes.

**Trial registration:**

German Clinical Trials Register, identification number DRKS00015313. Registered on 1 October 2018.

## Background

The risk of suffering from a subsequent anterior cruciate ligament (ACL) injury following ACL reconstruction is increased 10-fold in comparison to the first-incidence risk. A recurrence risk of 10–25% is specified in the current literature [1, 2]. Such re-injuries often occur in the first years following the surgery [2], in particular during, or shortly after, the successful return to sport (RTS). It is a major goal of the RTS process to lead an athlete back to training and competition without exhibiting an excessively high risk for a subsequent rupture [3]. Important criteria to be fulfilled, beyond psychosocial readiness and (of course) morphological graft healing, are, particularly, the restoration of neuromuscular and motor function. More precisely, a combination of dynamic strength and jump landing tests have been shown as being predictive for a second ACL injury [4, 5]. Enhancing and, thus, restoring these functional abilities may consequently lead to a decrease in the re-injury risk [6]. Beyond these quantitative measures, the quality of dynamic jumping and cutting maneuvers, in particular the avoidance of a dynamic valgus position, are crucial for a positive RTS decision based on functional criteria [7, 8]. Qualitative assessments are used only infrequently [7, 8]. Furthermore, their responsiveness to functional changes as well as predictive value for a subsequent ACL injury remains mostly unknown. A current systematic review with meta-analyses on biomechanics during single-leg landings concludes that both movement quality and performance of functional tasks should be examined as RTS tests [9]. Irrespective of the evaluation goal or if they having been validated or not, RTS tests are commonly performed at one single assessment at the hypothetical end of the RTS process [10]. Performing a series of measurements with the purpose of monitoring changes over time during the RTS process may be more promising [10].

As strength and dynamic motor-control capacities, in particular, are preventive for a re-injury [4, 5], they have to be targeted in recurrence prevention programs. Inadequate knee mechanics also result from trunk displacement and unequal limb loading [11]; thus, it is not sufficient to only target the knee joint. As an important surrogate of functional restoration of the knee and, likewise, not only triggered by the knee joint solely, avoiding a dynamic knee valgus may be crucial to prevent subsequent injury [11, 12]. Although contextual overlap between therapeutic and preventive programs (such as the described dynamic motor-control enhancement and avoidance of a dynamic knee valgus) exists, the therapeutic focus after ACL reconstruction should shift from therapy to recurrence prevention after a certain rehabilitation time. Several prevention programs exist and have been validated in terms of preventive effects on a primary ACL rupture incidence [13]. Although theoretical evidence exists that such programs may be effective in the prevention of a subsequent ACL injury [6], their effectiveness for primary and, in particular, for secondary prophylaxis after successful ACL reconstruction, is still a matter of debate. Likewise, the implementation capacity of such programs into everyday training after the prescribed rehabilitation is unknown.

The aim of the study is to assess the effects and implementation capacity of a preventive approach (Stop-X program [14]) after ACL reconstruction. Effects on re-injury-related motor-control/function, re-injury rates and time to successful RTS form the focus of this study.

The null hypothesis is as follows: the Stop-X program, as part of the evidence-based care and rehabilitation after ACL rupture and reconstruction, is not systematically superior to standard rehabilitation in terms of its effects on neuromuscular function, re-injury rate risk and time until RTS success.

## Methods and design

### Study design**,** ethics and informed consent

A multicenter, single-blind, randomized controlled, superiority, two-arm trial will be adopted. The comparator arms are guideline rehabilitation plus Classic follow-up treatment versus guideline rehabilitation plus the Stop-X intervention.

Central ethical approval has been confirmed from the independent Ethics Committee of the Hessen Regional Medical Council (ref approval no. FF 104/2017) and we will not begin recruiting at other centers in the trial until local ethical approval has been obtained. The date of the central approval was 27 June 2018. The study was planned and is performed in agreement with the Declaration of Helsinki (Version Fortaleza 2013). The trial was registered in the German Clinical Trials Register (DRKS), registration number DRKS00015313 (DRKS, drks.de; 01. October 2018). All adverse events will be reported. Each participant signs an informed consent prior to study enrollment.

The primary outcome is the normalized knee-separation distance (%) during a standardized Drop Jump Screening Test (DJST).

### Participants

#### Sample size determination

No study to retrieve the minimal clinical relevant change for the primary outcome DJST has been published. For the sample size calculation (G*Power version 3.1), we thus considered the values from an interventional randomized controlled trial (RCT) [12]. The mean post-intervention value was 66.4% (standard deviation (SD) = 14.2%). Considering a target effect of Cohen’s *d* = 0.3 (= mean change of 0.3 × SD), a two-sided alpha-error level of 5% and beta-error level of = 50%; data from *N* = 174 (*n* = 87 per group) participants have to be analyzed to statistically find a between-group difference using gain-score analyses. Assuming a dropout-rate of 30%, a total of 250 (*n* = 125 per group) patients will be included.

#### Recruitment and screening

Potential participants are recruited by personal addressing through one of the center heads (physicians) during or after a scheduled visit. Following application of the inclusion and exclusion criteria, patients are approved by the physician in charge.

Inclusion and exclusion criteria:

Adults (aged at surgery from 18 to 35 years) with remaining anterior knee instability after acute unilateral ACL rupture and having passed, or being scheduled for, an arthroscopically applied, anatomic single reconstruction (< 3 months between rupture and reconstruction) with autologous hamstrings tendon, will be included. Only participants engaged in sport (self-reported) prior to the injury and with the aim to return to their previous sporting activity are included. Exclusion criteria for the multimodal assessment protocol are: (1) meniscus lesion > 2 cm, (2) cartilage lesion > International Cartilage Regeneration and Joint Preservation Society (ICRS) grade II, (3) previous musculoskeletal surgery of the uninvolved (contralateral) leg, (4) leg mal-alignment > 5°, (5) multi-ligament injury pattern, (6) postoperative re-injury, (7) acute or chronic inflammation of the musculoskeletal system or muscle soreness and (8) pregnancy.

#### Randomization procedure and blinding

After sequential inclusion, volunteers are block-randomized using a non-stratified, permuted block with varying length strategy (*N* = 15 blocks; patient distribution = 1:1 in each arm/group. Each study sites’ participants are randomized using the site-specific randomization lists; the randomization sequence is generated using a computer-based algorithm (www.randomisation.com). All assessors and study personnel other than one study coordinator (DN) are blinded to group allocation. Participants are told not to communicate their group allocation to other participants or study staff. All data assessed are processed and analyzed blinded to treatment allocation.

### Experimental procedure

#### Study flow

**Fig. 1:**
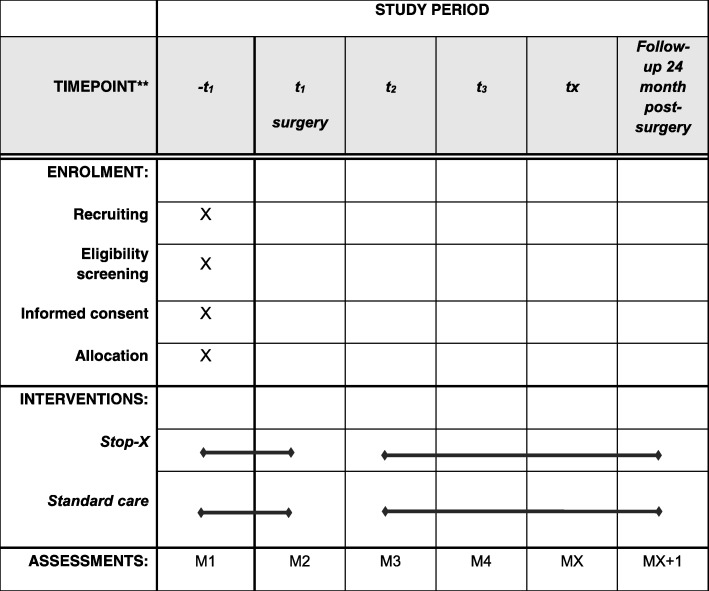
Standard Protocol Items: Recommendations for Interventional Trials (SPIRIT) flowchart: Schedule of enrollment, interventions and assessments

The onset of the Stop-X program, as a part of (where applicable) the pre-operative and/or the postoperative follow-up treatment, is individualized: a graduated, shared and criteria-based decision is underlying [10]. The participants must be released for the training components by their treating orthopedic specialist and by their physiotherapists. Likewise, the athletes, themselves, must indicate their physiological readiness. It is aimed to commence the Stop-X program at a time 4 to 8 months post surgery (Fig. 1).

The end of the (scheduled) intervention is the unrestricted RTS decision (aimed at 12–18 months post surgery). After this time point, the participants may continue the intervention but no further functional assessments will be conducted. Before (where applicable) surgery and until the unrestricted RTS decision, but at least until 12 months post surgery, all outcomes are assessed once a month. Thus, each participant is measured at least five times to a maximum of 12 times. Twelve (12), 18 and 24 months post reconstruction, follow-up measurements and recurrence monitoring follow. For this purpose, all participants are contacted by means of a structured telephone interview (separate consent). Participants are then asked for potential subsequent ACL re-injuries and other complaints/injuries. All measurements, except the structured telephone interviews, are performed by self-administration. The measures consist of jump landing tasks (10 min duration) and a questionnaire battery (5 min duration). The DJST is followed by the functional secondary outcomes. The latter consists of quality assessments during simple (combined) balance side and front hops, as well as quantity (centimeters) assessments of single-leg hops for distance. All hop/jump tests are self-administered, filmed from the frontal view (3-m distance) using the participant’s own smartphone. Videos are than expert-rated using the blinded videos. This setup has been shown to be valid when compared to 3D motion-capture systems for the analyses of sagittal-plane knee angles [15]. All videos are transferred using a safe big content transfer (PowerFolder Enterprise File Sync und Share; Düsseldorf; Germany). To assure blind assessment, only the participants’ pseudonym is used.

Secondary patient-reported outcomes are assessed questionnaire-based: knee function, kinesiophobia, RTS after ACL injury and training/therapy volume (frequency – intensity – type and time). All questionnaires are completed online at www.surveymonkey.de using the participant’s pseudonym.

Both the training and measurements are instructed and supervized by means of (online) control of training, outcome assessment adherence and correct execution.

#### Primary outcome – Drop Jump Screening Test

The DJST is a common RTS criteria with a predictive value on an ACL rupture [8, 16]. Following warm-up exercises, patients take a bipedal hip-width stance on a box with a 32-cm target height. A bipedal drop jump follows: frontal step – drop – reactive jump with the shortest possible ground contact time. The knee position is rated at three pre-defined points during this drop-jump cycle: (1) initial ground contact at the end of the drop from the box, (2) lowest point of the body’s center of gravity at the jump’s reversal point and (3) ground take-off when the feet leave the surface for the reactive vertical jump. At each of these points, the distance between (a), the hip joints and (b), the centres of the patella of the two knees are measured. The percentage of the knee distance in comparison to the hip distance was calculated to calculate the normalized knee distance. Knee-separation and hip distance is analyzed using the video-analysis software Kinovea (France). Three jumps are performed, the middle one to be further analyzed. This measurement setup and the subsequent analyses are highly inter-rater reliable (κ = .92; 95% confidence interval (CI) 0.829–0.969) and moderately intra-rater reliable (κ = .55; 95% CI 0.49–0.61) [17], respectively. In the same validation study, a sensitivity of 63% and a specificity of 83% for the classification of “high-risk” participants (index group identified by expert observers) of the DJST was found.

#### Secondary outcomes: quality and quantity of the functional hop and jump tests

For sagittal-plane jumping-quality rating, the single Balanced Front Hop Test [18] was chosen. Participants have to hop over a square on the floor with a 40 × 40-cm edge length with hands on their hips; the end position after landing must be kept for at least three continuous seconds. The frontal-plane jumping-quality rating is undertaken using the Balanced Side Hop Test [18]; the participants hop over laterally (to the direction of the test foot (right or left) ) the same square and, immediately after landing, a reactive hop back to the starting position is initiated. After each trial, the participants have, again, to maintain the controlled landing position for three consecutive seconds with their hands on their hips [18].

Three hops for each condition and leg (randomized order) are performed, the middle one for each condition and leg to be further analyzed. Quality rating criteria are (1) feet remain stable on the ground after landing, (2) knees remains in the sagittal plane during all movements and (3) the trunk remains in the sagittal plane during all movements [18].

The single-leg hop for distance (SLHD) assesses (the quantity of) dynamic balance and single-leg-jumping performance ability. The participants stand on one leg with the toes behind the rear line of the square. They then hop as far as possible and have to land in a controlled manner. The whole hop must be performed one-legged. Three successful hops per leg (randomized order) are performed; each leg’s best trial (in centimeters) is selected for further analysis. The measurement properties for the SLHD are excellent, reliability is ICC = 0.97 (CI 0.9–0.99) and the standard error of measurement is 3.5% [19]. It is considered valid in terms (as a major part of a testing battery) of predicting a subsequent ACL injury [4, 5].

#### Secondary outcomes: self-reported data

All questionnaires will be used in their validated German translation.

The Return-to-Sport after ACL injury (RSI-ACL) questionnaire assesses a participants’ fear of loading their reconstructed knee during their main sporting activity. Ten 11-point Likert-scale questions are used, a total score value of > 56 indicates sufficient confidence for RTS [20]. The sum score assessment is highly reliable (Cronbach’s alpha = 0.92) [21] and valid [22].

Further (self-reported) control variables to be used will be the Tegner and Lysholm score, International Knee Documentation Committee knee evaluation form (IKDC-2000), Knee injury and Osteoarthritis Outcome Score (KOOS), subscale activities of all daily living (ADL), injury history, demographics, pain, and task-specific fear of movement/re-injury (visual analog scale (VAS) 10 cm) and kinesiophobia (Tampa Scale of Kinesiophobia; TSK).

The Tegner and Lysholm score [23] contains a 0–10-point Likert scale to assess a participant’s activity level, scaled from low-level daily living activity to high-level competitive sports level. Scoring is from 0 = low-level activity regarding knee loading, up to and including 10 = highest possible level of activity regarding knee loading. The Lysholm knee scoring scale rates symptoms and function during ADLs using eight scales to build a 0–100-point sum score. Test-retest reliability was found to be sufficient [22]. The IKDC-2000 uses 18 items across three subdomains to assess: symptoms, sports activities and function. Differently graded Likert scales are used. Test-retest reliability is high, whereas the internal consistency and cross-cultural validity are unknown [22]. The KOOS subscale ADL is one of five KOOS subdomains and must be scored from 0 to 4. Test-retest reliability was found to be high [22]. The TSK uses 11 items across the domain “fear of movement/(re-)injury”; it is scored using 4-point Likert scales. Internal consistency and structural validity were found to be sufficient [22].

By means of a structured telephone interview, the time/date of the consensus, evidence-based, shared, positive return-to-play decision and potential re-injuries/complications/adverse effects are inquired. Further, the participants will be asked on their individual implementation capacity (barriers and chances) using a non-structured survey.

### Intervention

The training interventions (in particular the Stop-X arm) consist of self-administered home-based exercises which are step-wise graduated and follow wound-healing and functional restoration criteria. The training frequency for both arms is scheduled to be three times per week. A duration of 30 min is aimed for each session. The program follows current (secondary) prevention guidelines [24–26]. The program (in German) can be found online (pdf): http://deutsche-kniegesellschaft.de/wp-content/uploads/2017/02/DKG_Stop-X_Prävention-von-Sportverletzungen-am-Kniegelenk.pdf (permanent link, status 10/5/18) and (video description): https://stop-x.de/(permanent link, status 10/5/18). Details on the two training schemes (Stop-X versus Classic) are displayed in Table 1. All aspects of the training are divided into the wound-healing phases and the therapy goal. All similarities of and differences between the Classic and Stop-X exercise regimens are displayed.

**Table 1:** Therapy/intervention regimen for both study arms. Standard/Classic rehabilitation strategies were conducted according to grade-A guidelines [27, 28]. The secondary preventive Stop-X program follows the recommendations in [24–26]

Wound-healing phase and/or time post surgery	Therapy goal	Classic	Stop-X
Inflammation phase	Reduction of inflammation	Brace: range of motion restriction 0–0-90° at 20-kg partial knee load	
		Manual therapeutic, passive closed kinematic chain mobilization; hip and ankle integrated	
		Measures against post-operative swelling and temperature increase, home-based measures for auto-mobilization (under compression)	
		Trunk and scapula control; non-affected-side static motor control, i.e., single-leg stance	
Proliferation phase Approximately weeks 3–12 post reconstruction	Pain reduction and mobilization [27, 29]	Knee-joint mobilization in restricted range of movement, patellar mobilization	
		Stretching: Mm. ischiocruales, M. gastrocnemius, M. iliopsoas, M. tractusiliotibialis	
		Motor control of trunk and scapula; non-affected-side static motor control, i.e., single-leg stance	
Approximately after week 7 post reconstruction		Brace: range of motion unrestricted	
		Onset of motor-control training	
		Open kinetic (dorsal only) chain	
Approximately after week 10 post reconstruction		Corrective exercise with the aim to prepare for return to sport (RTS) level I	
		Single-leg dynamic-stabilizing/motor-control exercises	
		Eccentric knee mobilization open frontal kinetic chain	
Remodeling phase Level I function: Approximately after week 13 post reconstruction	Functional enhancement and recurrence prevention	Closed and open kinetic chain (ventral and dorsal)	Based on functional criteria, not time based. Information, risk assessment, correction of risky movement characteristics; teaching of basic preventive strategies; total program at/after RTS clearance Running exercises/agility exercises Straight on running, hip external rotation, change of direction runs Running with sagittal plane distance and vertical-jump components. Self-perturbed balance exercises Single-leg stance with (individually and combined) ball bouncing/unstable surface/passing a ball Jumping exercises/plyometrics vertical and distance Strengthening exercises Russian hamstrings Dynamic and side planks with/without heel raising Hip abduction One-legged squats
		Strength endurance training for (in particular) the ischiocrural muscles	
		Dynamic-stabilizing motor-control exercises, including jump landings on unstable surfaces	
Level II function: Approximately months 4–6 post reconstruction		Dynamic-stabilizing motor-control exercises, including single (affected) leg jump landings on unstable surfaces	
		Onset of impact exercises	
Level III function: Approximately after month 7 post reconstruction		Dynamic exercises (frontal plane)	
		Side-cutting maneuvers	
Level IV function: Not before 10th month post reconstruction		Dynamic multi-directional stabilization	

All participants receive detailed information on the intervention scheduled. Classic intervention participants receive a standard follow-up scheme with defined key points; the Stop-X participants receive the same Classic intervention scheme and, additionally, the detailed information in terms of information material and specific instructions (print material and links) for the Stop-X program. All participants discuss their program with the therapy team (physiotherapists, athletic trainer).

### Therapy monitoring

To monitor home-training accuracy and compliance, all patients fill in an exercise log during the home-based training (online). Frequency, intensity, type and time of the exercises are reported in a standardized procedure. Likewise, all functional/exercise measures beyond the scheduled training are reported. Using the 14-step Likert scale of Borg rates of perceived exhaustion (6 = extremely light intensity; 20 = extremely vigorous), the intensity of the exercises is further rated.

### Statistical analysis

Following range plausibility control based on potentially reachable physiological values, a visual control for non-plausible outliers will be performed. Afterwards, all analyses will be performed based on the underlying assumptions for parametric, or rather, non-parametric testing. In detail, for all difference testing, data and variance distribution (i.e., normality) are checked; for all correlation analyses, residuals, auto correlation, and heteroscedasticity, are checked. The alpha-error threshold is set at 5%, all *p* values below are considered significant.

The analyses for the primary outcome, DJST, are done confirmatory, alpha-error corrections (Bonferroni-Holm for paired and Sidak-Holm for unpaired analyses) are performed for multiple used samples and post-hoc analyses following significant omnibus testing, whilst the alpha-error remains unadjusted for the exploratory (secondary) analyses.

The main statistics are performed twice (two different data structures): once on a time-based and once on a functional-based data structure. All repeated measures statistics are thus first performed for the time-based data structure where statistics are performed on all outcomes assessed at the same real-time after reconstruction. Secondly, functional-based data-structure analyses are performed, where the first (baseline) measurement is the first “real” measurement at the time of being cleared for the assessment (varying times of 4–8 months post reconstruction). For each of the analyses on the two datasets, gain-score analyses are performed for differences from pre (baseline) to post (each following time point) of all outcomes using repeated-measures analyses of co-variance (rmANCOVAs) with potential confounders as co-variates (baseline values, injury history, sex, type and amount of sport, age, co-morbidities). In case of a systematic impact of confounders on statistics, z-transformed values are calculated and used for further analyses. Afterwards, survival analyses by means of Kaplan-Meier plotting plus between group-difference analyses using log-rank tests are performed. Hazard ratio estimations will be calculated. Events will be: (1) Time point of unrestricted RTS decision, (2) Time point of functional criterion RTS succeeded (> 60% normalized knee-separation distance at DJST) and (3) Time point of revision surgery, re-injury or subsequent injury.

All statistical analyses are performed using SPSS (SPSS Inc., IBM, Chicago, IL, USA) and BiAS (BiAS for Windows, Frankfurt, Germany).

## Discussion

The primary aim of the study is to test the feasibility and effectiveness of an ACL secondary preventive intervention. To do so, a standardized diagnostic assessment and a randomized controlled, single-blind intervention design are used. The intervention consists of exercises to target deficits in motor control and dynamic knee axis steadiness. A strength of this intervention is its practicability. Furthermore, all exercises can be performed without (or only with a little) additional material.

Recurrence prevention and the according diagnostics during and after RTS following ACL reconstruction are, except in professional athletes, only adopted to a minor degree [30]. Even in professional sports, the athletes’ performance quality is decreased after return to competition [1] and the re-injury risk remains high for years after the ACL rupture [1, 2]. Consequently, and applicable in entire populations, developing and employing strategies for secondary prevention and appropriately adapted functional diagnostics are crucial. Beyond the focus on recurrence prevention, the potential of the program in pre-operative training may be given. Pre-operative training strategies are highlighted to be of importance [31], yet, they are only carried out sparsely; therefore, a feasible intervention may be helpful.

The heterogeneity of the participants may become a limitation for the results’ interpretation and the deduction of the practical relevance of our trial. To minimize this potential bias, co-variate analyses are performed. Together with the randomized study design and the resulting respect of unknown confounders, considering the known and suggested confounders provides a sufficient statistical power. A common limitation in clinical exercise trials is the limited possibility of blinding the patients. This limitation is reduced by the superiority design (no inactive control group) of our study. To further reduce the risk of bias as much as possible, all investigators are blinded to intervention allocation.

Beyond this, the results of our study will be of importance not only for researchers and policy makers, but also for patients.

## Trial status

Participant recruiting started on 25 October 2018. At the time of manuscript submission, we included six participants into the study. No participant has yet completed the intervention. Screening completion is anticipated to be 15 October 2019, measurement completion by 15 October 2020 and overall study completion is expected to be May 2021.

## Data Availability

Not applicable.

## Appendix

### Study sites, local principal investigators and scientific responsibilities

Principal investigator:

PD Dr. Dr. Stein, Thomas, Department of Sporttraumatology, Knee, and Shoulder Surgery, Berufsgenossenschaftliche Unfallklinik Frankfurt am Main, Frankfurt am Main, Germany

Co-principal investigator, statistics:

Dr. Niederer, Daniel, Department of Sports Medicine, Goethe University Frankfurt, Frankfurt am Main, Germany

Prof. Dr. Dr. Banzer, Winfried, Department of Preventive and Sports Medicine, Institute for Occupational, Social and Environmental Medicine, Goethe University Frankfurt, Frankfurt, Germany

Functional outcomes:

Keller, Matthias, OSINSTITUT, Munich, Germany

Recruitment centers:

PD Dr. Achtnich, Andrea, Department for Orthopaedic Sports Medicine, Klinikum rechts der Isar, Munich, Germany

Dr. Akoto, Ralph, Chirurgisch-Traumatologisches Zentrum, Asklepios Klinik St. Georg, Hamburg, Germany

Prof. Dr. Ateschrang, Atesch, BG Trauma Center Tübingen, Eberhard Karls University Tübingen, Tübingen, Germany

Dr. Barié, Alexander, Clinic for Orthopedics and Trauma Surgery, Center for Orthopedics, Trauma Surgery and Spinal Cord Injury, Heidelberg University Hospital, Heidelberg, Germany

Dr. Best, Raymond, Department of Orthopaedic and Trauma Surgery, Sportsclinic Stuttgart,; Department of Orthopaedic Sportsmedicine, University of Tuebingen

Dr. Ellermann, Andree, Arcus Sportklinik, Pforzheim, Germany

Prof. Dr. Marzi, Ingo, Prof. Dr. Frank, Johannes, Department of Trauma, Hand, and Reconstructive Surgery, Goethe University Frankfurt, Frankfurt am Main, Germany

Prof. Dr. Herbort, Mirco, Department of Trauma, Hand and Reconstructive Surgery, University Hospital Muenster, Muenster, Germany

Prof. Dr. Höher, Jürgen, Sports Clinic Cologne at Cologne Merheim Medical Center, Cologne, University of Witten/Herdecke, Cologne, Germany

PD Dr. med. Daniel Guenther, Department of Orthopaedic Surgery, Trauma Surgery, and Sports Medicine, Cologne Merheim Medical Center, Witten/Herdecke University

Dr. Jung, Tobias M, Center for Musculoskeletal Surgery, Charité-University Medicine Berlin, Berlin, Germany

PD Dr. Krause, Matthias, Department of Trauma, Hand and Reconstructive Surgery, University Medical Center Hamburg-Eppendorf, Hamburg, Germany

Prof. Dr. Müller, Peter E; Andreas Fischer, Department of Orthopedic Surgery, Physical Medicine and Rehabilitation, University Hospital, Ludwig-Maximilians-University (LMU), Munich, Germany

Prof. Dr. Petersen, Wolf, Klinik für Orthopädie und Unfallchirurgie, Berlin, Germany

Dr. Stoffels, Thomas, Department of Trauma and Orthopaedic Surgery, Unfallkrankenhaus Marzahn, Berlin, Germany

Dr. Stöhr, Amelie, Orthopedic Surgery Munich, Munich, Germany

Dr. Welsch, Frederic, Department of Sporttraumatology, Knee, and Shoulder Surgery, Berufsgenossenschaftliche Unfallklinik Frankfurt am Main, Frankfurt am Main, Germany

## Abbreviations

ACL: Anterior cruciate ligament
ADL: Activities of daily living
DJST: Drop Jump Screening Test
DRKS: German Clinical Trials Register
IKDC-2000: International Knee Documentation Committee knee evaluation form
KOOS: Knee injury and Osteoarthritis Outcome Score
RTS: Return to sport
SD: Standard deviation
SLHD: Single-leg hop for distance
TSK: Tampa Scale of Kinesiophobia
